# Erythematous scalp papules and alopecia in a middle-aged woman

**DOI:** 10.1016/j.jdcr.2025.06.036

**Published:** 2025-07-07

**Authors:** Lauren Fleshner, Mehmet Fatih Atak, Katie Roster, Frederick Pereira, Kenneth Shulman, Banu Farabi

**Affiliations:** a School of Medicine, New York Medical College, Valhalla, New York; b Dermatology Department, NYC Health + Hospitals/Metropolitan, New York, New York; c Dermatology Department, NYC Health + Hospitals/Coney Island, Brooklyn, New York; d Dermatology Department, Icahn School of Medicine, Mount Sinai Hospital, New York, New York

**Keywords:** alopecia, breast, cancer, cutaneous metastasis, scalp

## Case description

**Fig 1:**
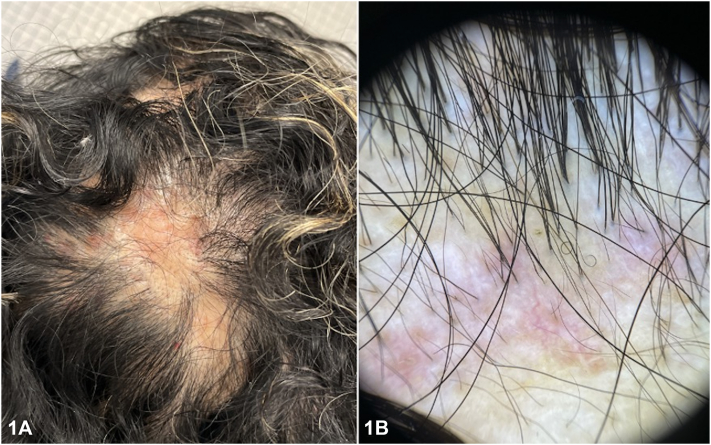

A 53-year-old female presented with hair loss and scalp pruritus persisting for over 1 year. Physical examination revealed cicatricial alopecic plaques with subtle erythema on the midline scalp (Fig 1, *A* and *B*) and a positive hair-pull test. Initial scalp biopsy revealed mild spongiosis and perivascular inflammation with eosinophils. After 6 weeks, the patient reported worsening hair loss, breathlessness, joint pain, and weight loss. Examination revealed new circular alopecic patches throughout the scalp, loss of follicular ostia, and new erythematous papules.

**Fig 2:**
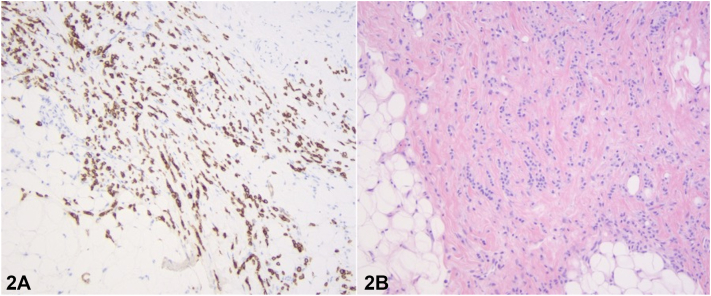

**Fig 3:**
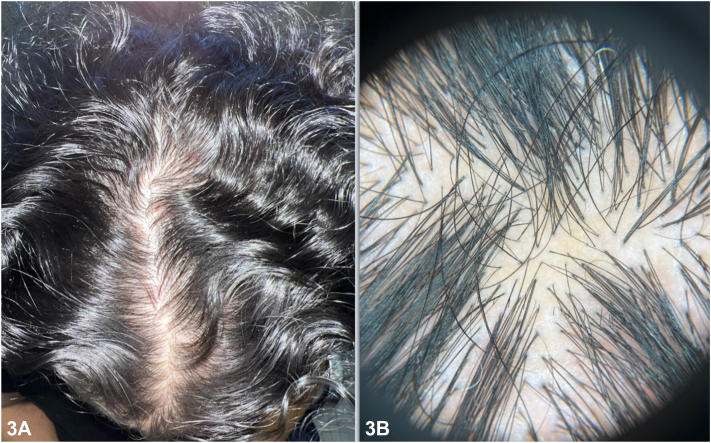

Three months later, the patient returned with notable scarring alopecia with erythema. Dermatoscopy revealed an erythematous background with telangiectasias and white lines with loss of follicular orifices. A second biopsy of an erythematous papule at this stage revealed dermal infiltration of atypical epithelial cells arranged in cords between fibrotic stroma (Fig 2, *A*). Immunohistochemically, cells were positive for cytokeratin 7 (Fig 2, *B*) and estrogen receptor. Treatment with 5% minoxidil solution was initiated. Follow-ups at 7 and 15 months revealed regrowth and regression of lesions, primarily in adjacent areas where some follicular ostia likely remained viable (Fig 3, *A* and *B*).

**Question 1: What is the most likely diagnosis?**

A. Metastatic breast cancer to the scalp
B. Alopecic and aseptic nodules of the scalp (AANS)
C. Central centrifugal cicatricial alopecia
D. Lichen planopilaris (LPP)
E. Discoid lupus erythematosus (DLE)

**Answers:**

A. Metastatic breast cancer to the scalp – Correct. Neoplastic alopecia (NA) is a rare presentation of cutaneous metastasis, a diagnosis that is often associated with breast cancer.^1^ In this case, scalp biopsy revealed dermal infiltration of atypical epithelial cells arranged in cords between fibrotic stroma, with immunohistochemical positivity for cytokeratin 7 and estrogen receptor, confirming the diagnosis.
B. Alopecic and aseptic nodules of the scalp (AANS) – Incorrect. AANS presents with 1 or more alopecic nodules and may mimic NA, especially when neoplastic cells form a nodular appearance. Clinical differentiation can be challenging, as AANS may also present with firm, asymptomatic scalp nodules. The noncicatricial nature of AANS may be appreciated with dermatoscopy. Histopathology of AANS shows a nonspecific mixed inflammatory cell infiltrate, which may form granulomas or pseudocysts.^2^
C. Central centrifugal cicatricial alopecia – Incorrect. Pink papules are commonly reported in NA, with erythema noted in almost half of cases.^1^ Pink papules and an erythematous background may help distinguish NA from other pathologies.^3^
D. Lichen planopilaris (LPP) – Incorrect. LPP is often associated with a lichenoid lymphocytic infiltrate on histology.
E. Discoid lupus erythematosus (DLE) – Incorrect. DLE typically presents with scarring alopecia associated with erythema, scaling, and follicular plugging. The biopsy in this case showed dermal infiltration by malignant epithelial cells without these features.

**Question 2: Which of the following clinical or histological findings most strongly suggest a diagnosis of neoplastic alopecia?**

A. Perifollicular lymphocytic inflammation with fibrosis
B. Brown halos surrounding hair shafts on trichoscopy
C. Infiltration of dermis by cytokeratin 7 positive epithelial cells
D. Loss of follicular ostia with mucin deposition on biopsy
E. Plasma cells and basement membrane thickening

**Answers:**

A. Perifollicular lymphocytic inflammation with fibrosis – Incorrect. This finding would most likely be found in inflammatory scarring alopecia.
B. Brown halos surrounding hair shafts on trichoscopy – Incorrect. Brown halos, also known as peripilar halos, are associated with androgenetic alopecia and telogen effluvium.
C. Infiltration of dermis by cytokeratin 7 positive epithelial cells – Correct. Cytokeratin 7 is an epithelial marker expressed by breast carcinoma cells and confirms NA.^4^
D. Loss of follicular ostia with mucin deposition on biopsy – Incorrect. This is a hallmark of follicular mucinosis, characterized by follicular papules or indurated plaques.
E. Plasma cells and basement membrane thickening – Incorrect. These features are seen in DLE, not NA.

**Question 3: What other cancers can metastasize to the scalp?**

A. Renal cell carcinoma
B. Gastrointestinal tract
C. Thyroid
D. Uterine
E. All of the above

**Answers:**

A. Renal cell carcinoma – Incorrect.
B. Gastrointestinal tract – Incorrect.
C. Thyroid – Incorrect.
D. Uterine – Incorrect.
E. All of the above – Correct.

Common primary tumors associated with scalp metastases originate mostly from the gastrointestinal tract, lungs, prostate, and breast.^5^

## Conflicts of interest

None disclosed.
